# Welcome to *Microbial Informatics and Experimentation*

**DOI:** 10.1186/2042-5783-1-1

**Published:** 2011-06-14

**Authors:** Michael J Wise, Barry L Wanner

**Affiliations:** 1 Biomolecular, Biomedical and Chemical Sciences, M310, University of Western Australia, Crawley WA 6009, Australia; 2 Department of Biological Sciences, Purdue University, 915 W. State Street, West Lafayette, IN 47907, USA

## Editorial

*Microbial Informatics and Experimentation (MIE) *is a journal about computers and microbes. We created a new journal to fill a gap for which there is no publication avenue that is particularly geared to computationally-oriented, strongly biologically motivated, pragmatic articles focused on microbes. On the one hand, the bioinformatics journals are generally very computer technical and unlikely to be read by the diverse community of microbiologists. There is also a strong emphasis in bioinformatics literature on human/mammalian systems, though this is a secondary issue. On the other hand, microbial informatics work has appeared in a variety of microbiological publications, but it is seldom a good fit there either, and methods that span diverse microbes have no obvious home.

We envisage the readership being practising microbiologists who are interested in computational insights into their data, and bioinformaticists interested in the microbial species and systems that sustain life on our fragile planet, but that also can make us very ill. The 1918 Influenza (Spanish flu) had an estimated death toll of 50-100 million [1], more than either the first or second World Wars. However, without microbes (starting with the symbionts that became mitochondria and chloroplasts), life as we know it would not exist today. A further dimension is the context in which our work is now taking place: climate change and changing land-use patterns due to the expanding world population, which will increasingly impact all species, starting with microbes. Given the complexity of processes now being studied, computers have joined PCR machines, agar plates and restriction enzymes, as tools for understanding the species and systems we investigate.

We see our topics of interest being as broad as the diversity of species we cover. A starting list of topics would include: microbial comparative genomics, microbial protein structure and function, microbial systems and metagenomics, microbial systems biology and the mathematical modelling of microbial systems. The list is not exhaustive, and will certainly evolve over time. If you have work at the interface between computation and microbiology (both broadly defined), we would be happy to consider it. Our aim for the journal, embodied in the refereeing processes, is that it be collegial and constructive. In a phrase, a community. Please join us.

Michael J Wise and Barry L Wanner, on behalf of The *Microbial Informatics and Experimentation *Team
